# Species richness and β-diversity patterns of macrolichens along elevation gradients across the Himalayan Arc

**DOI:** 10.1038/s41598-021-99675-1

**Published:** 2021-10-11

**Authors:** Subzar Ahmad Nanda, Manzoor-ul Haq, S. P. Singh, Zafar A. Reshi, Ranbeer S. Rawal, Devendra Kumar, Kapil Bisht, Shashi Upadhyay, D. K. Upreti, Aseesh Pandey

**Affiliations:** 1 Department of Botany, University of Kashmir, Sriangar, Jammu & Kashmir 190006 India; 2 Central Himalayan Environment Association, Dehradun, India; 3 G. B. Pant National Institute of Himalayan Environment and Sustainable Development (GBPNIHESD), Kosi-Katramal, Almora, 263 643 India; 4 G.B. Pant National Institute of Himalayan Environment and Sustainable Development, Sikkim Regional Centre, Pangthang, Gangtok, 737 101 India; 5 National Botanical Research Institute, 436, Rana Pratap Marg, Prem Nagar, Hazratganj, Lucknow, Uttar Pradesh 226001 India

**Keywords:** Ecology, Plant sciences, Ecology

## Abstract

Understanding the species richness and β-diversity patterns along elevation gradients can aid in formulating effective conservation strategies particularly in areas where local anthropogenic stresses and climate change are quite significant as in the Himalaya. Thus, we studied macrolichen richness and β-diversity along elevational gradients at three sites, namely Kashmir (2200 to 3800 m a.m.s.l), Uttarakhand (2000–3700 m a.m.s.l) and Sikkim (1700 to 4000 m a.m.s.l) which cover much of the Indian Himalayan Arc. In all, 245 macrolichen species belonging to 77 genera and 26 families were collected from the three sites. Only 11 species, 20 genera and 11 families were common among the three transects. Despite the differences in species composition, the dominant functional groups in the three sites were the same: foliose, fruticose and corticolous forms. The hump-shaped elevation pattern in species richness was exhibited by most of the lichen groups, though an inverse hump-shaped pattern was also observed in certain cases. β-diversity (β_sor_) based on all pairs of comparisons along an elevation gradient varied from 0.48 to 0.58 in Kashmir, 0.03 to 0.63 in Uttarakhand and 0.46 to 0.77 in Sikkim. The contribution of turnover to β-diversity was more than nestedness at all the three transects. Along elevation β-diversity and its components of turnover and nestedness varied significantly with elevation. While species turnover increased significantly along the elevation in all the three transects, nestedness decreased significantly in Kashmir and Sikkim transects but increased significantly in the Uttarakhand transect. Except for the Kashmir Himalayan elevation transect, stepwise β-diversity and its components of turnover and nestedness did not vary significantly with elevation. The present study, the first of its kind in the Himalayan region, clearly brings out that macrolichen species richness, β-diversity, and its components of turnover and nestedness vary along the elevation gradients across the Himalayan Arc. It also highlights that contribution of turnover to β-diversity is higher in comparison to nestedness at all the three transects. The variations in species richness and diversity along elevation gradients underpin the importance of considering elevational gradients in planning conservation strategies.

## Introduction

Elevation gradients in mountains with co-varying microclimatic conditions and varied exposure, aspect, topography, natural and anthropogenic disturbances, and other environmental factors provide microhabitats and ecological niches for a wide variety of species^1–3^. Relationship between species richness and elevation have been widely studied^4,5^, and monotonic, unimodal or multimodal patterns have been observed^6^. Several authors have reported mid gradient peaks (Mid Domain Effect) in species richness^7–10^. Unlike many such studies, far less is known about β-diversity and its components of turnover and nestedness in relation to elevation. The studies so far carried out have mostly shown a decline in β-diversity with increasing elevation^11^ and variable contributions from its components of turnover and nestedness. The turnover component, a measure of the difference in species composition between two or more species assemblages, is a consequence of environmental filtering or spatial and historical constraints^12^ while nestedness component (one assemblage is a subset of another) may either be a function of the number of niches available or occupied at different sites in a study area^13^ or due to extinctions in poor sites and/or colonizations in rich sites along the gradients^14^. Environmental filtering (where the environment selects against certain species) is also expected to contribute to nestedness^15^ by filtering only a subset of species along large spatial extents. Very few studies have analysed variation in these two components of β-diversity along elevational gradients^16–19^ and the available results suggest that species turnover may decrease and nestedness may increase with elevation, though such patterns may differ in different regions^18^. Many ecologists, however, argue that β-diversity along elevational gradients is primarily caused by species turnover and have suggested that species sorting and abiotic changes are the main drivers of these elevational species replacements^19^. Thus, the study of β-diversity patterns can provide important insights into understanding the mechanisms of community assembly along environmental or geographic gradients, and also in the context of climate change^20,21^. Such studies are particularly relevant to the Himalaya which is a biodiversity hotspot and is experiencing climate change and other anthropogenic stresses. But not many studies related to the patterns of species richness, β-diversity and its components of turnover and nestedness along elevation gradients have been carried out so far.

To fill this knowledge gap, we carried out the present study on macrolichens along three elevation transects that represent much of the east to west Himalayan Arc^22–26^. We selected macrolichens for several reasons such as, easy identification, a rich assemblage of about 2,300 species belonging to 305 genera and 74 families growing in India^27^, their occurrence over a wide elevation range varying from 0 to 7400 (m a.m.s.l.)^28^ and sensitivity to climate. In addition, different groups of lichens are reported to show distinct elevational patterns in species richness^28–32^. The present multilocational and comprehensive macroecological investigation is expected to provide deeper insights into the richness of macrolichens and β-diversity patterns along the elevational gradients across the Himalayan arc. We also hypothesized that β-diversity and its turnover component would decline and nestedness would increase with elevation.

## Methods

### Study area

The present study was undertaken at three elevational transects in Kashmir (North-western Himalaya), Uttarakhand (Central Himalaya) and Sikkim (Eastern Himalaya) in the Indian Himalayan Region (IHR). The details of the three transects are given in Table 1. In the IHR region, annual precipitation increases several-fold and temperatures get warmer as one moves from the west to the east. While Uttarakhand and Sikkim sites are strongly monsoonal with 70–80% annual precipitation occurring from June to September, the Kashmir site has weak monsoon (~ 50% of annual precipitation), and precipitation is mostly in the form of snow during winter. In Sikkim and Uttarakhand sites, forests are mixed having both conifers and broadleaved species; in Kashmir, the forests are dominated by conifers but the dominant treeline species is broadleaved *Betula utilis* (birch). In Uttarakhand transect, evergreen oaks (*Quercus floribunda* and *Q. semecarpifolia*) dominate most of the elevation gradient, and oaks occur also in treeline along with fir (*Abies spectabilis*) and birch (*Betula utilis*). Forests in Sikkim are moist and diverse with several rhododendron species. *Abies densa* is the main treeline conifer. *Q. lamellosa* is the main oak (evergreen), but generally occurs mixed with several other species. Oaks are absent in Kashmir.

**Table 1:** Geographical location and other characteristics of elevation transects.

Study transect	Elevation range	Geographical coordinates	Vegetation type	Climate
Daksum- Sinthan Top, Kashmir (DSTK)	2200–3800 (m a.s.l.)	33°36′43"N and 75°26′6"E 33°34'N and 75°30'E	Mixed evergreen coniferous forest with dominance of *Abies pindrow, Pinus wallichiana, Picea smithiana,* and broadleaved *Betula utilis* as the main treeline species	The climate in Kashmir is sub-Mediterranean type with four seasons (spring, summer, autumn, and winter) based on mean temperature and precipitation. Kashmir Himalaya experiences an annual minimum temperature of about 7.3 °C and annual maximum temperature of about 19.7 °C with annual precipitation of about 1000 mm
Tungnath Timberline Landscape, Uttarakhand (TTLU)	2000–3700 (m a.s.l.)	30°29′22″N and 79°12′55″E	The dominant forest vegetation includes evergreen broadleaf forests of *Quercus semecarpifolia*, and *Rhododendron arboreum*; evergreen needle-leaf coniferous forests—*Abies pindrow*, and *A. spectabilis*; and Krummholz of *R. campanulatum*	Climate of Uttarakhand is monsoonal with dry pre- monsoon. Temperature and precipitation varies considerably across the region. At Tungnath (study site) the annual mean temperature ranges between 17.2 °C (at 1600 m a.s.l.) to 6.0 °C (at 3680 m a.s.l.). Annual precipitation ranges between 2209–2598 mm. However, pre-monsoon precipitation varies between 280–380 mm annually
Khangcehndzonga National Park, Sikkim (KNPS)	1700–4000 (m a.s.l.)	27°42′N 88°08′E	The dominant vegetation includes evergreen broadleaf forests of *Lithocarpus pachyphyllus*, *Castanopsis histrix, Quercus lamellosa, Rhododendron arboretum, R. hodgsonii*; evergreen needle leaf coniferous forests of *Tsuga dumosa* and *Abies densa* leading to krummholz thickets and alpine scrubs dominated by *Rhododenron* spp. and *Juniperus* spp.	The climate of Sikkim state varies extremely due to high elevation and ranges from sub-tropical in south to tundra in the north. The area falls under monsoonal precipitation regime and based on monsoon circulation the state possess four prevalent seasons viz. (i) cold (Dec–Feb), (ii) Spring (March–May), (iii) Monsoon (June–Sep), (iv) Period of retreating monsoon (Oct–Nov). In Yuksam-Dzongri transect the mean temperature ranges between 14.45 °C (at 1700 m a.s.l.) to 4.25 °C (at 4000 m a.s.l.). Annual precipitation ranges between 765 and 1021.0 mm

**Figure 1:**
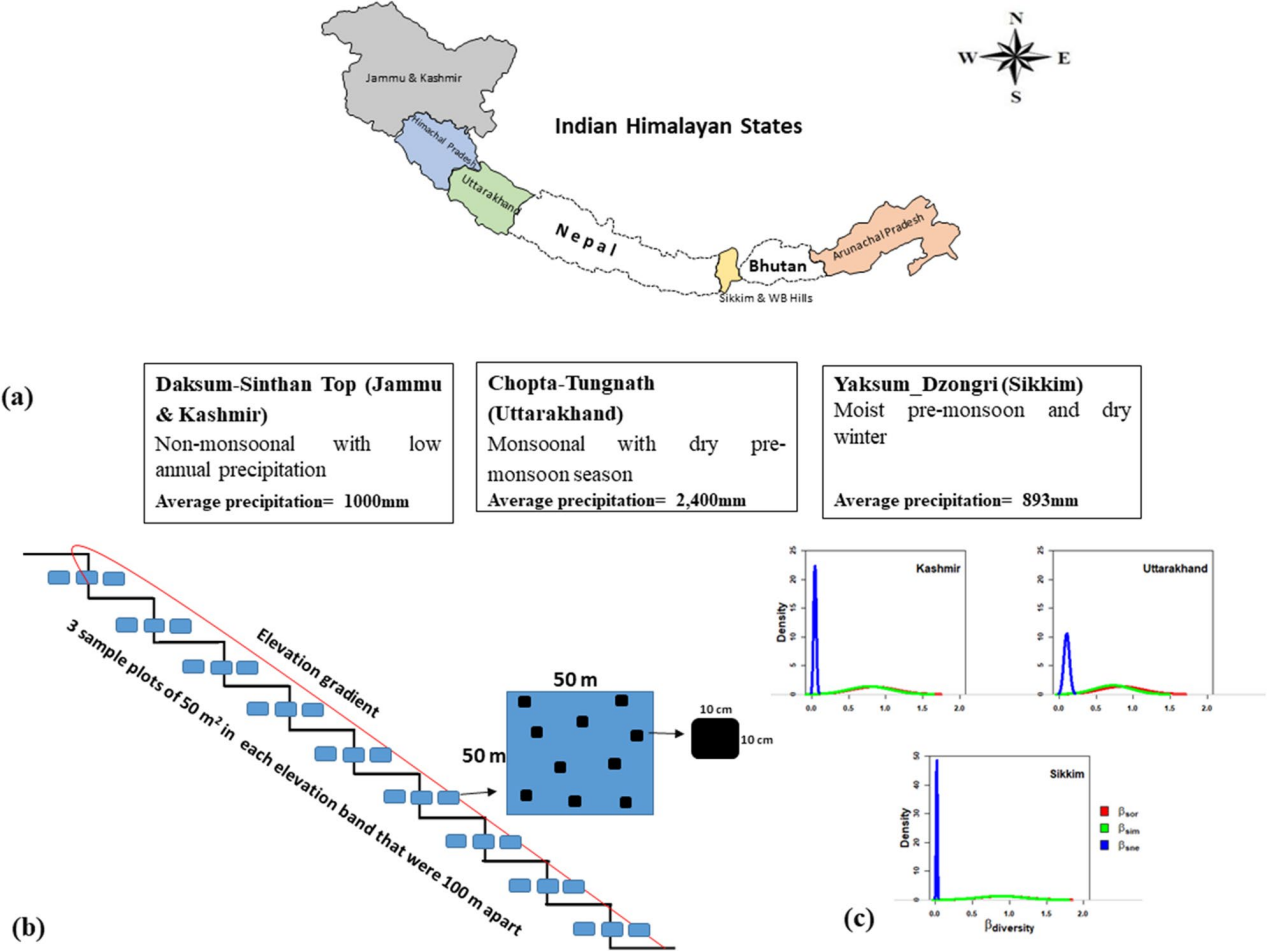
Location of the study sites and a schematic view of the study design (**a**,**b**). β-diversity (β_sor_) of macrolichens based on all possible pair-wise comparisons, its components of turnover (β_sim_) and nestedness ((β_sne_) were studied along the entire elevation gradient at the three sites.Moreover,, along elevation β-diversity based on pairs starting from the lowest elevation band to all other elevation bands and stepwise β-diversity based on pairs from one elevation band to the next neighbouring elevation band were also computed (**c**).

Each transect was divided into elevational bands that were 100 m apart. The number of elevational bands in the Kashmir, Uttarakhand and Sikkim transects was 17, 18 and 24, respectively. Three plots of 50 × 50 m area were established in each of these elevational bands. In each plot, the habitats, such as soil, rocks, tree trunks etc. were surveyed thoroughly for the collection of macrolichens using the stratified sampling method. Ten quadrats of 10 cm^2^ were laid for each of the available habitat types in each of the three 50 m^2^ plots per elevational belt (see also^33^) to collect lichen samples from May to October during 2017–2019 (Fig. 1).

The collected lichen specimens were air-dried, curated, and examined according to the standard lichenological procedures^34^. Samples were morpho-anatomically examined under a stereomicroscope (Leica S8 and Leica DM 500). Relevant keys and monographs^35–39^ were used for proper identification. The identified and authenticated specimens were deposited at the National Botanical Research Institute, Lucknow (LWG) and the Department of Botany, University of Kashmir, Srinagar, India (KASH).

### Data analyses

We first verified our sampling completeness by estimating sampling coverage with the function iNEXT using the R package *inext*^40^. Datatype was set to “incidence_freq” since species presence was used and not the abundance. Species richness was calculated by computing the cumulative number of lichen species per site. A non-metric multidimensional scaling (NMDS) based on Bray–Curtis dissimilarity values was used to determine the distance between the different sites using the metaMDS function in the R package *vegan*^41^. We used the spline.plot function within the R package *drsmooth*, to plot a spline estimated dose–response function on the actual richness data along the elevational gradient with its upper and lower 95 per cent confidence bounds. β-diversity was calculated using the occurrence data which was then partitioned into turnover and nestedness components by applying the function beta.pair within the R package *betapart*^42^. This resulted in three matrices based on pair-wise comparisons of each site: the Sørensen dissimilarity index (β_sor_) that expresses the total compositional variation with values ranging between 0 and 1, the Simpson dissimilarity index matrix (β_sim_) that represents the compositional changes due to species turnover, and β_sor_ minus β_sim_ is the resultant nestedness component (β_sne_). We further processed β-diversity data (β_sor_, β_sim_, β_sne_) to distinguish between (i) along elevation β-diversity accounting for pairs starting from the lowest elevation to all other elevations, and (ii) stepwise β-diversity including pairs from one elevation to the next neighbouring elevation band^43^. All these analyses were carried out using R version 3.6.2 (R Core Team, 2019). The effect of elevation on both along gradient as well as stepwise β-diversity was tested with a linear mixed-effects model using the Ordinary Least Squares (OLS) Regression method in the Past software (version 4.02)^44^.

## Results

### Species estimation curves

**Figure 2:**
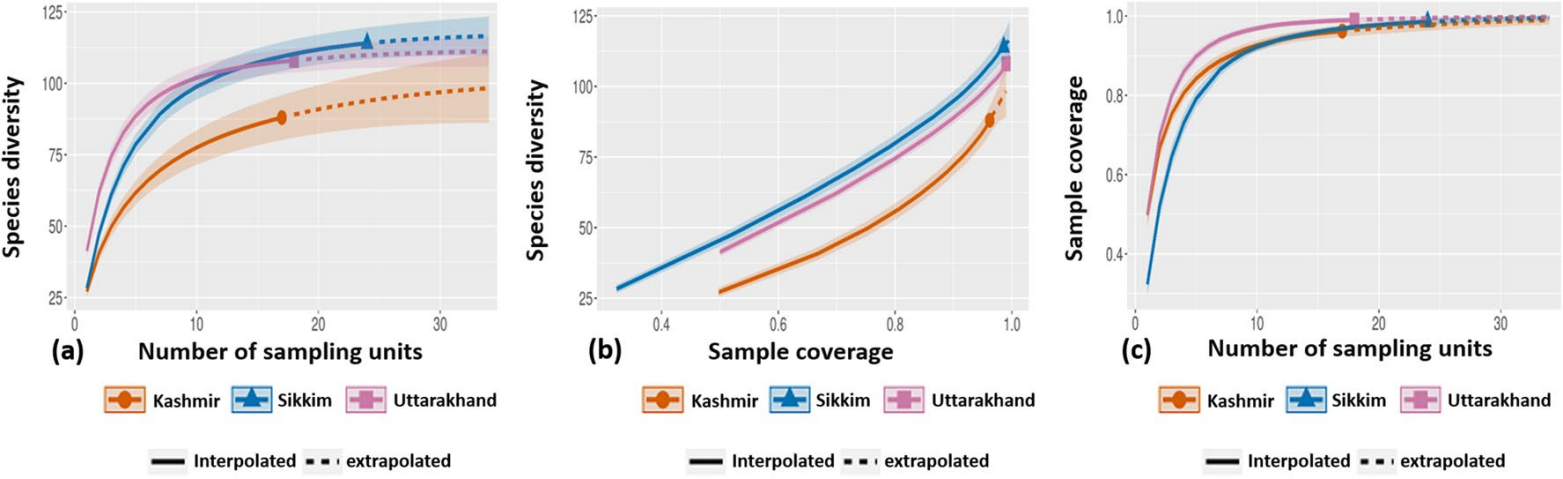
Species estimation curves: **(a)** sample-size-based rarefaction/extrapolation sampling curves **(b)** coverage-based rarefaction/extrapolation sampling curves and **(c)** sample completeness curves based on sample size and sample coverage for three elevation transects with different sample sizes (red colour—17 samples in Kashmir, purple colour—18 samples in Uttarakhand and blue colour—24 samples in Sikkim). The shaded portion in **(a)** represents the upper and lower bound 95% confidence intervals to the estimated richness. The cumulative number of lichen species (*y*-axis) is plotted as a function of the cumulative number of samples (*x*-axis) pooled in a random order.

The sample-size-based rarefaction/extrapolation curves which plot the diversity estimates in relation to sample size reached an asymptote (Fig. 2a) indicating that the sampling was adequate in all three regions. The coverage-based R/E sampling curves (Fig. 2b) which plot the diversity estimates with respect to sample coverage revealed an increase in species diversity with the sample completeness (as measured by sample coverage) and a small slope in the sample completeness curve before its extrapolation indicated sufficient sample size (Fig. 2c).

### Taxonomic and functional diversity

**Figure 3:**
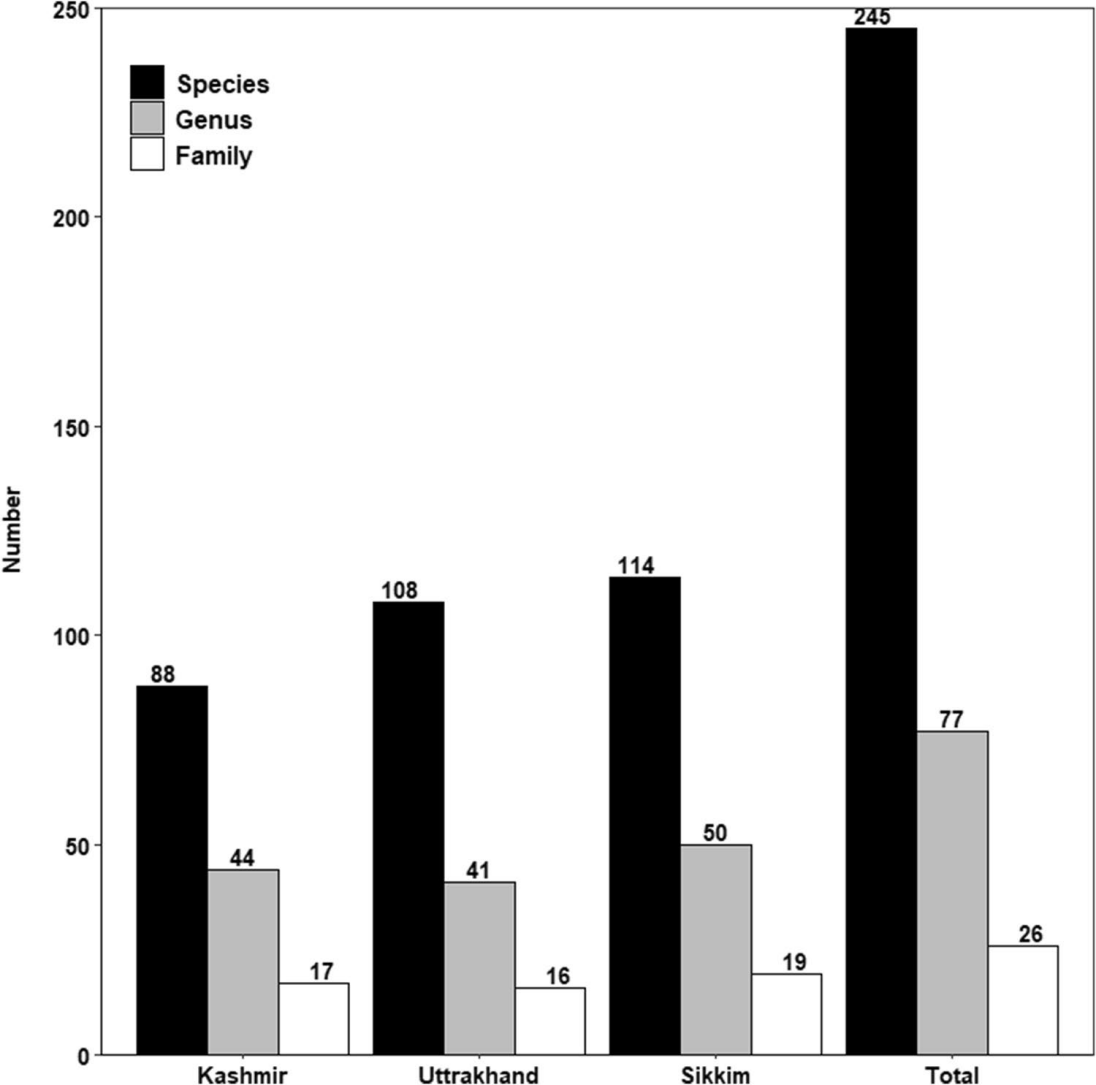
Taxonomic conspectus of macrolichens in Kashmir, Uttarakhand and Sikkim Himalaya.

A total of 245 macrolichen species belonging to 77 genera and 26 families were documented (Fig. 3) from the three elevation transects in the Indian Himalayan Region (IHR).

**Figure 4:**
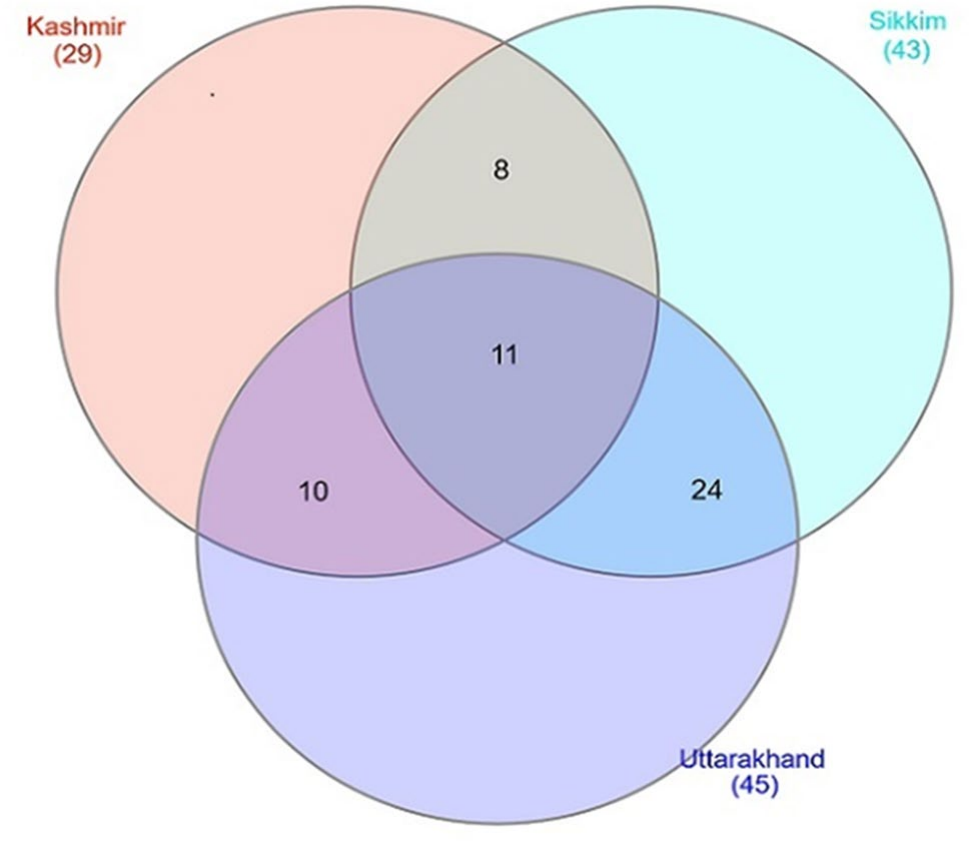
Venn diagram showing the number of lichen species shared between the three transects.

Eighty-eight macrolichen species were recorded in the Kashmir Himalayan transect, 108 in Uttarakhand and 114 in Sikkim. Only 11 species, 20 genera and 11 families were common among the three transects. Ten species were common between Kashmir and Uttarakhand, eight between Kashmir and Sikkim and twenty-four species were common between Uttarakhand and Sikkim (Fig. 4).

**Figure 5:**
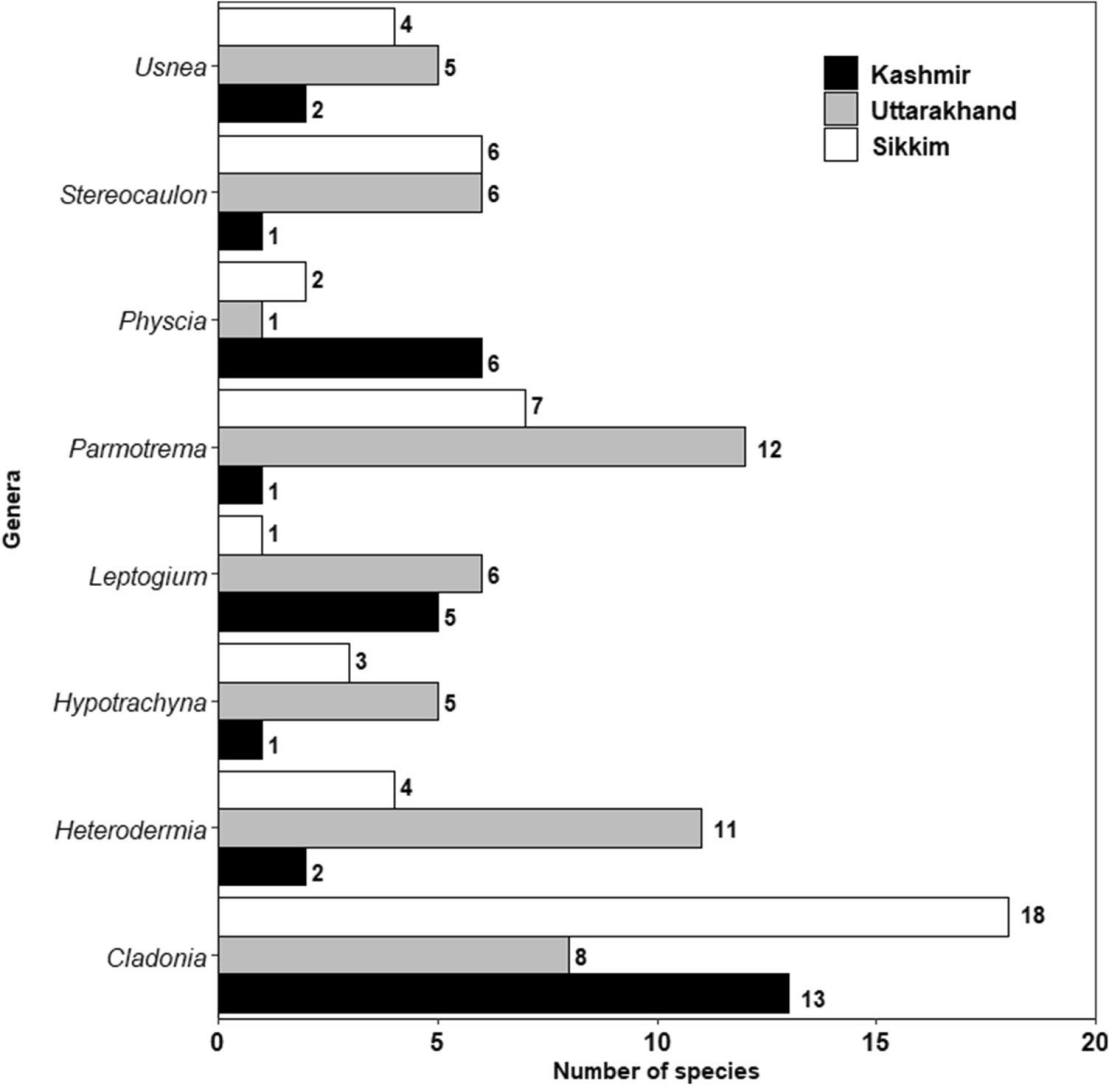
Eight dominant genera of macrolichens in Kashmir, Uttarakhand and Sikkim.

**Figure 6:**
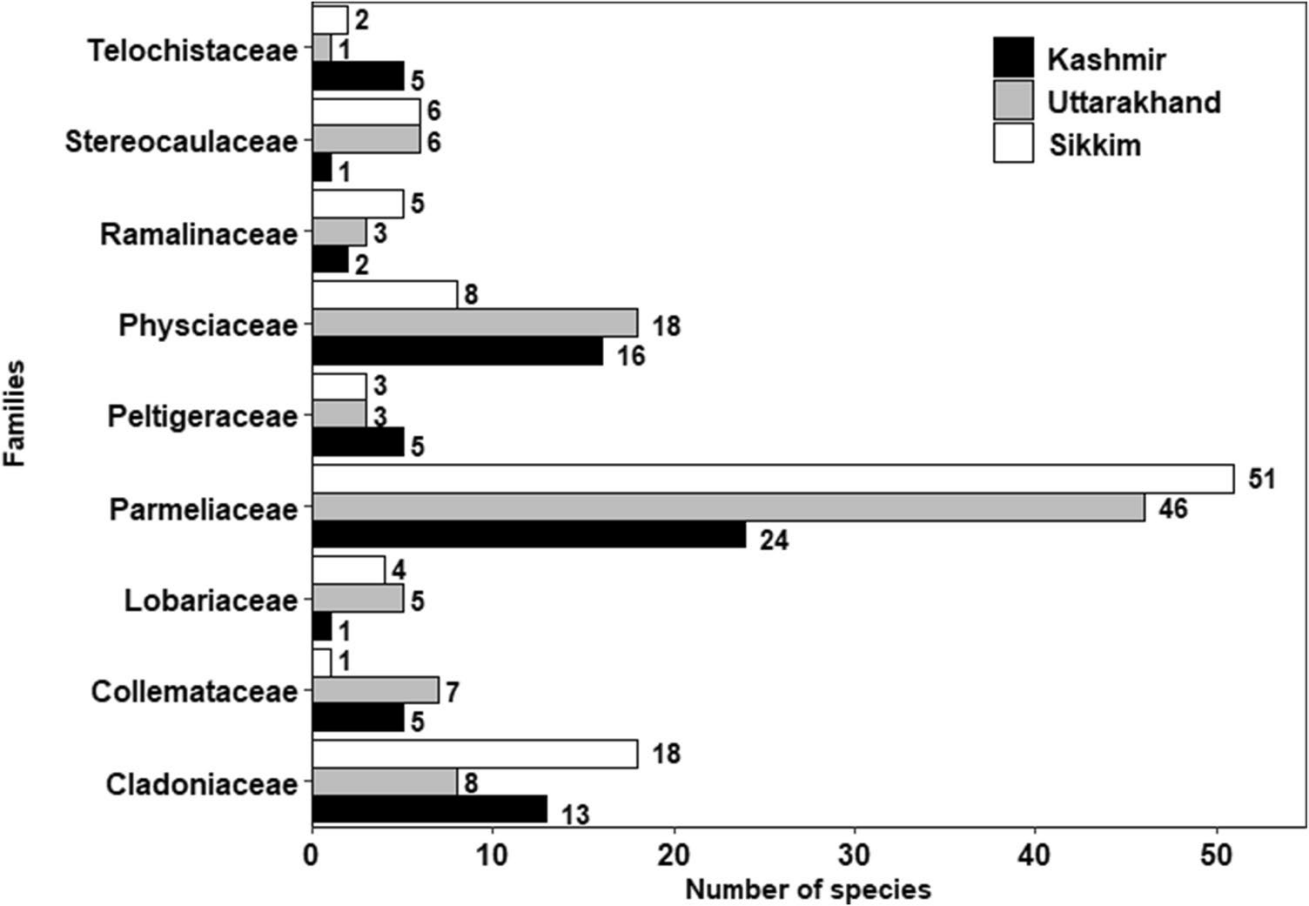
Nine largest families of macrolichens in Kashmir, Uttarakhand and Sikkim.

In respect of functional groups, foliose forms were the most abundant and were represented by 56 species in Kashmir, 86 in Uttarakhand and 66 in Sikkim followed by fruticose type with 7 species in Kashmir, 14 in Uttarakhand and 21 in Sikkim (Table 2). Among the macrolichens characterized on the basis of the substrate they inhabit, corticolous forms (growing on tree bark) were most common with 46 species recorded from Kashmir, 41 from Uttarakhand and 34 from Sikkim followed by saxicolous forms represented by 19 species in Kashmir, 15 in Uttarakhand and 21 in Sikkim. Terricolous lichens had 12 species in Kashmir, 15 in Uttarakhand and 26 in Sikkim. The relative number of macrolichen species that grew on both tree bark and rocky substrates (corticolous-saxicolous), bark and wood (terricolous-lignicolous), tree bark and soil (corticolous-terricolous) and in all the three habitats (corticolous-saxicolous-terricolous) is given in Table 2. *Cladonia* was the dominant genus represented by 13, 08 and 18 species in Kashmir, Uttarakhand and Sikkim, respectively. Parmeliaceae was the dominant family represented by 24, 46, 51 species in Kashmir, Uttarakhand and Sikkim, respectively (Figs. 5, 6).

**Table 2:** Number of macrolichen species belonging to various growth forms and habitat categories in Kashmir, Uttarakhand and Sikkim.

Growth form	Number of species		
	Kashmir	Uttarakhand	Sikkim
Crustose	8	0	8
Foliose	56	86	66
Fruticose	7	14	21
Squamulose	17	8	19
**Habitat categories**			
Corticolous	46	41	34
Saxicolous	19	15	21
Terricolous	12	15	26
Lignicolous	4	0	0
Lignicolous/Terricolous	2	0	0
Corticolous/ Ramulicolous	0	0	1
Corticolous/Saxicolous	4	19	15
Corticolous/Terricolous	1	5	4
Terricolous/Muscicolous	0	0	4
Corticolous/Saxicolous/Terricolous	0	10	7
Terricolous/Muscicolous/Saxicolous	0	0	1
Saxicolous/Terricolous	0	3	1

**Figure 7:**
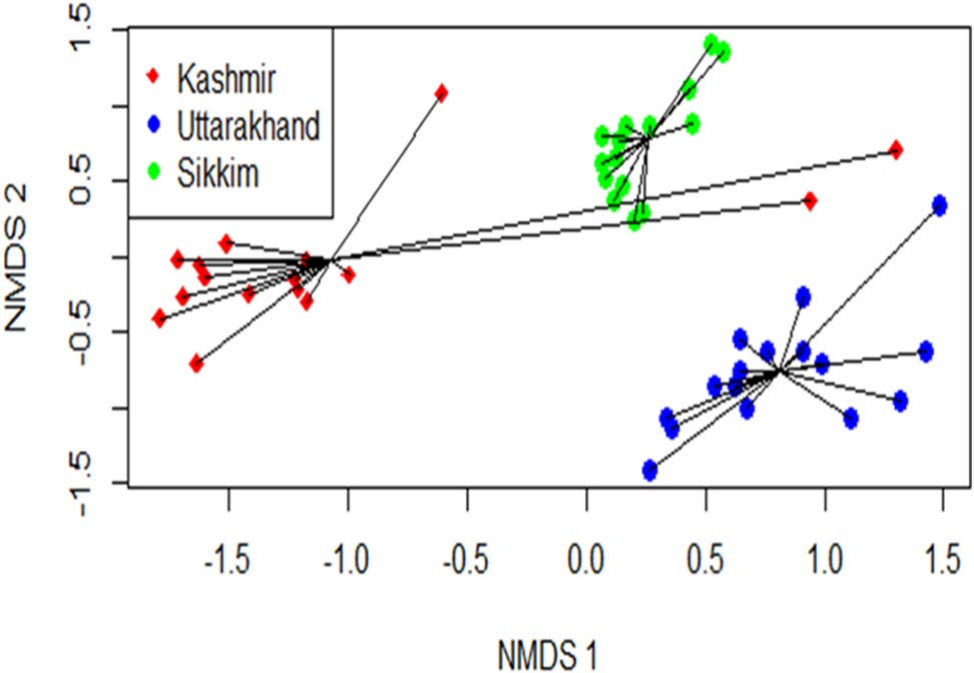
NMDS based on Bray–Curtis dissimilarity index for three elevation transects. The graphical plot represents the species compositions of three transects over 16 common elevation bands (communities) in an elevation span of 2200–3700 (m a.m.s.l.).

In a non-metric dimensional scaling (NMDS) ordination, three distinct clusters got aligned along the two-dimensional axis of NMDS plot in which communities (elevation bands) across the Sikkim transect showed more overlap than those in Kashmir and Uttarakhand (Fig. 7). In all, the three transects differed significantly in terms of their species composition (P < 0.001) using permutational multivariate analysis of variance (PERMANOVA) performed with 999 permutations (Table 3).

**Table 3:** PERMANOVA test results (n = 999) based on Bray–Curtis dissimilarity matrix for three elevation transects.

	Df	Sum of squares	Mean square	Model	R^2^	Pr(> F)
Transect	2	7.2945	3.6473	19.417	0.46323	0.001***
Residuals	45	8.4528	0.1878		0.53677	
Total	47	15.7473			1	

***(p ≤ 0.001).

### Elevation pattern in species richness

**Figure 8:**
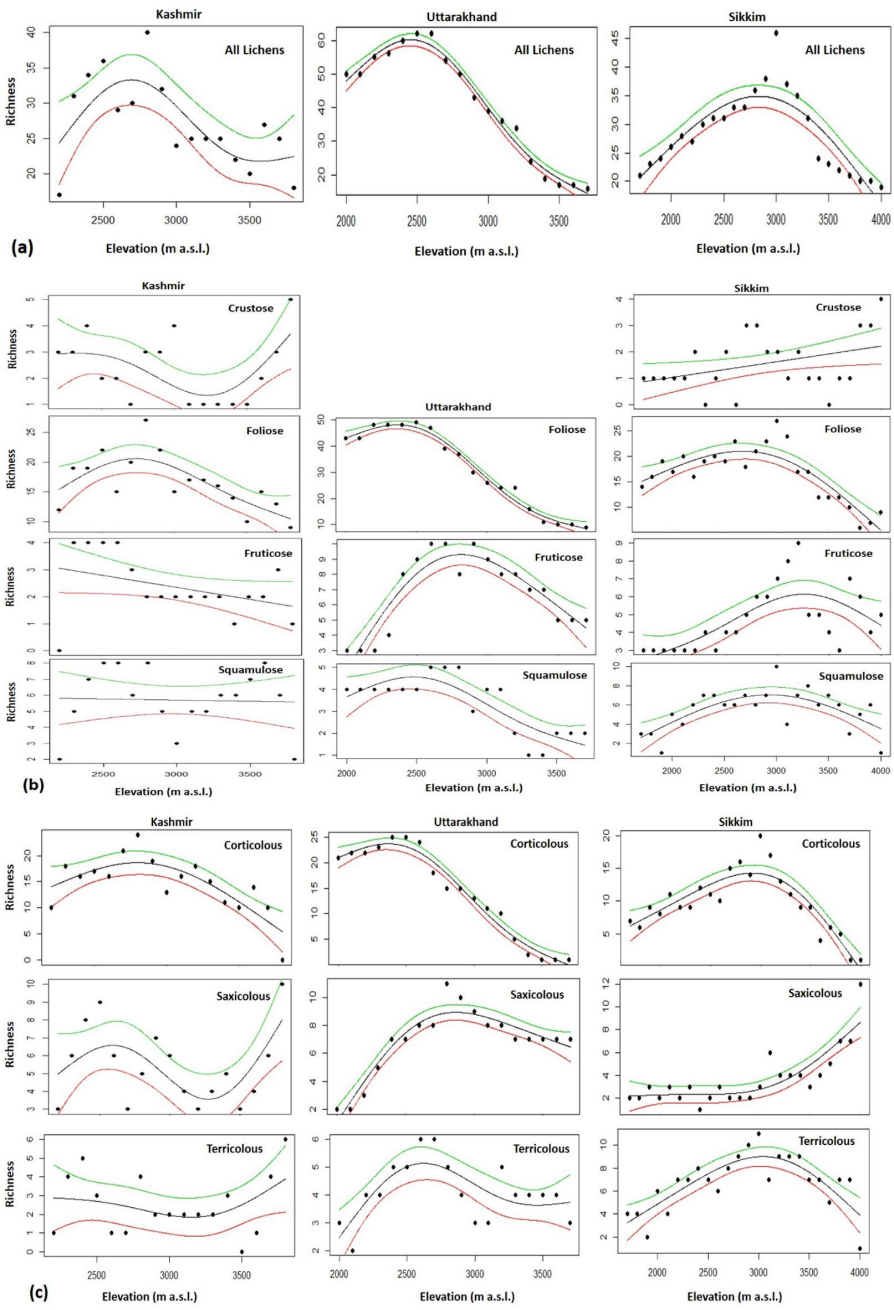
Relationship between species richness and elevation. **(a)** Total lichen richness, **(b)** functional groups and **(c)** habitat groups across three different regions of Kashmir, Uttarakhand and Sikkim. The middle black line represents the actual richness, upper (green line) and lower (red line) represent the 95 per cent confidence intervals.

The spline.plots based on total macrolichen species along the elevation gradient displayed a hump-shaped pattern in Kashmir and Sikkim while a low plateau type pattern was observed in Uttarakhand (Fig. 8). A more or less similar pattern was seen in the case of foliose and corticolous lichens. Hump shaped pattern was also noticed in fruticose and squamulose forms in Uttarakhand and Sikkim transects. Inverse hump-shaped patterns were observed for crustose and saxicolous forms in Kashmir. However, some lichen forms, such as fruticose, squamulose and terricolous in Kashmir and crustose in Sikkim did not reveal any significant relationship with elevation (Fig. 8).

### β-diversity

The mean total β-diversity based on pairs of comparisons between the elevation bands varied from 0.48 to 0.58 in Kashmir, 0.03 to 0.63 in Uttarakhand and 0.46 to 0.77 in Sikkim (Table 4). The narrow range in Kashmir indicated that all the elevation pairs differed more or less equally in lichen species composition. It was not true for Uttarakhand where some pairs had low β-diversity (more or less similar species composition) while others differed significantly in species composition (β-diversity of 0.63). The highest β-diversity value of 0.77 was recorded in Sikkim which indicated that a less number of species was shared between the two successive elevational bands. The mean turnover ranged from 0.36 to 0.54 in Kashmir, from 0.04 to 0.56 in Uttarakhand and from 0.41 to 0.76 in Sikkim. The trend in turnover was more or less similar to β-diversity and hence it is the turnover component that contributed more to β-diversity in comparison to nestedness which varied from 0.02 to 0.15 in Kashmir, from 0.02 to 0.28 in Uttarakhand and from 0.01 to 0.13 in Sikkim (Table 4). Lower values of nestedness indicated that the lichen assemblages of species-poor elevation bands were not sub-sets of the species in the species-rich elevation bands.

**Table 4:** Mean (± standard deviation) of β-diversity (β_sor_) and its turnover (β_sim_) and nestedness (β_sne_) components in Kashmir, Uttarakhand and Sikkim Himalayan transects.

β-diversity and its components	Kashmir	Uttarakhand	Sikkim
β_sor_	0.52 ± 0.04 (0.48–0.58)	0.41 ± 0.20 (0.03–0.63)	0.68 ± 0.06 (0.46–0.77)
β_sim_	0.42 ± 0.12 (0.36–0.54)	0.25 ± 0.21 (0.04–0.56)	0.61 ± 0.15 (0.41–0.76)
β_sne_	0.07 ± 0.04 (0.02–0.15)	0.14 ± 0.08 (0.02–0.28)	0.05 ± 0.03 (0.01–0.13)

Values in parentheses represent the range.

**Figure 9:**
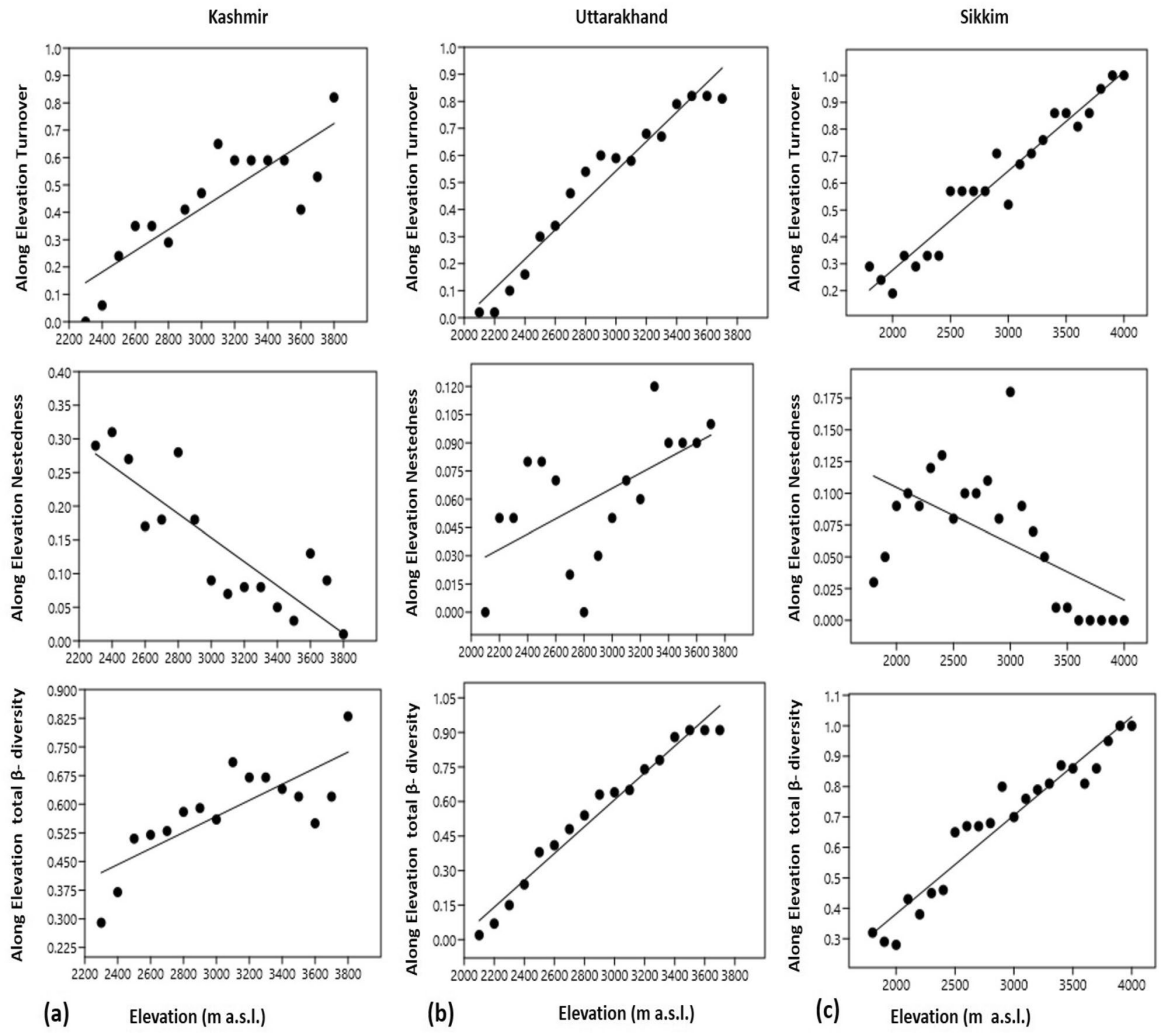
Turnover, nestedness and total β-diversity along the elevational transect: the effect of elevational distance on turnover, nestedness and total β-diversity was tested with a linear mixed-effects model. The line indicates the response of turnover, nestedness and overall β-diversity to elevational distance and the points display their values of pair-wise comparisons.

**Figure 10:**
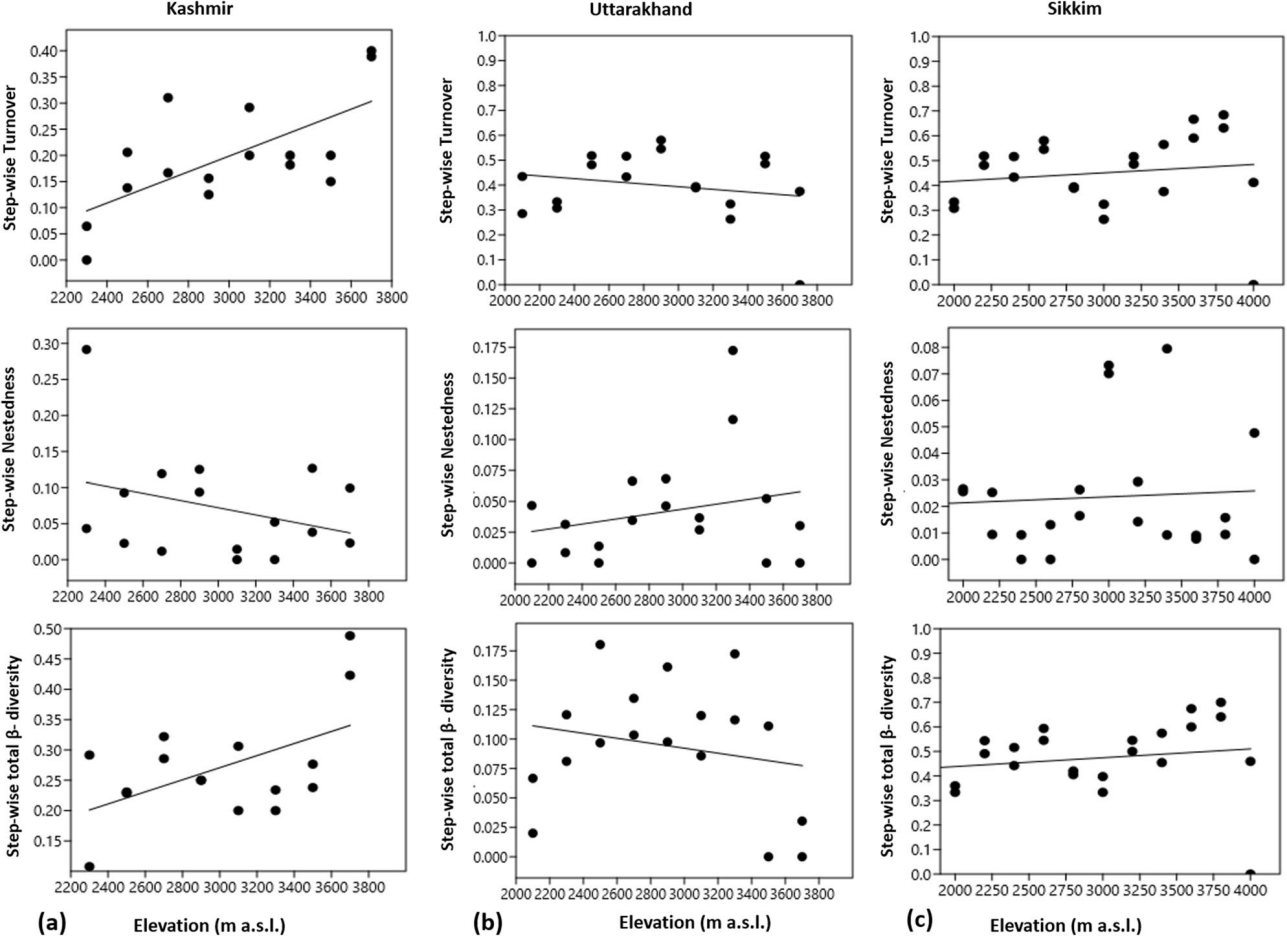
Stepwise turnover, nestedness and overall β-diversity: the effect of elevational distance on turnover, nestedness and overall β-diversity was tested with a linear mixed-effects model. The line indicates the response of turnover, nestedness and overall β-diversity to elevational distance and the points display their values of pair-wise comparisons.

Along elevation total β-diversity and turnover increased significantly (Table 5) with elevation in all the three transects (Fig. 9). Nestedness, however, decreased significantly with elevation in Kashmir and Sikkim but increased significantly (Table 5) in Uttarakhand (Fig. 9). A study of the stepwise β-diversity data (Fig. 10 and Table 5) revealed an insignificant relationship between total β-diversity, turnover and nestedness except for total β-diversity and turnover in Kashmir Himalaya which increased significantly with elevation (Fig. 10).

**Table 5:** Results of linear effect models testing the effect of elevational distance for overall β-diversity (β_sor_) and the components turnover (β_sim_) and nestedness (β_sne_) performed separately for along elevation and stepwise β-diversity.

		r	r^2^	t value	p value
**Along elevation β-diversity**					
Kashmir	β-diversity	0.7817	0.6111	4.6900	0.0003
	Turnover	0.8770	0.7691	7.0676	0.0000
	Nestedness	-0.8533	0.7281	-6.1227	0.0000
Uttarakhand	β-diversity	0.9839	0.9680	21.3160	0.0000
	Turnover	0.9731	0.9468	16.3430	0.0000
	Nestedness	0.6095	0.3715	2.9774	0.0094
Sikkim	β-diversity	0.9645	0.9302	16.7330	0.0000
	Turnover	0.9699	0.9408	18.2630	0.0000
	Nestedness	-0.5874	0.3451	-3.3262	0.0032
**Stepwise β-diversity**					
Kashmir	β-diversity	0.5306	0.2815	2.3421	0.0345
	Turnover	0.6652	0.4424	3.3330	0.0049
	Nestedness	-0.3192	0.1019	-1.2603	0.2282
Uttarakhand	β-diversity	-0.2063	0.0426	-0.8433	0.4115
	Turnover	-0.2076	0.0431	-0.8489	0.4085
	Nestedness	0.2417	0.0584	0.9965	0.3338
Sikkim	β-diversity	0.1733	0.0300	0.8254	0.4180
	Turnover	0.1558	0.0243	0.7398	0.4673
	Nestedness	0.0694	0.0048	0.3263	0.7473

**Figure 11:**
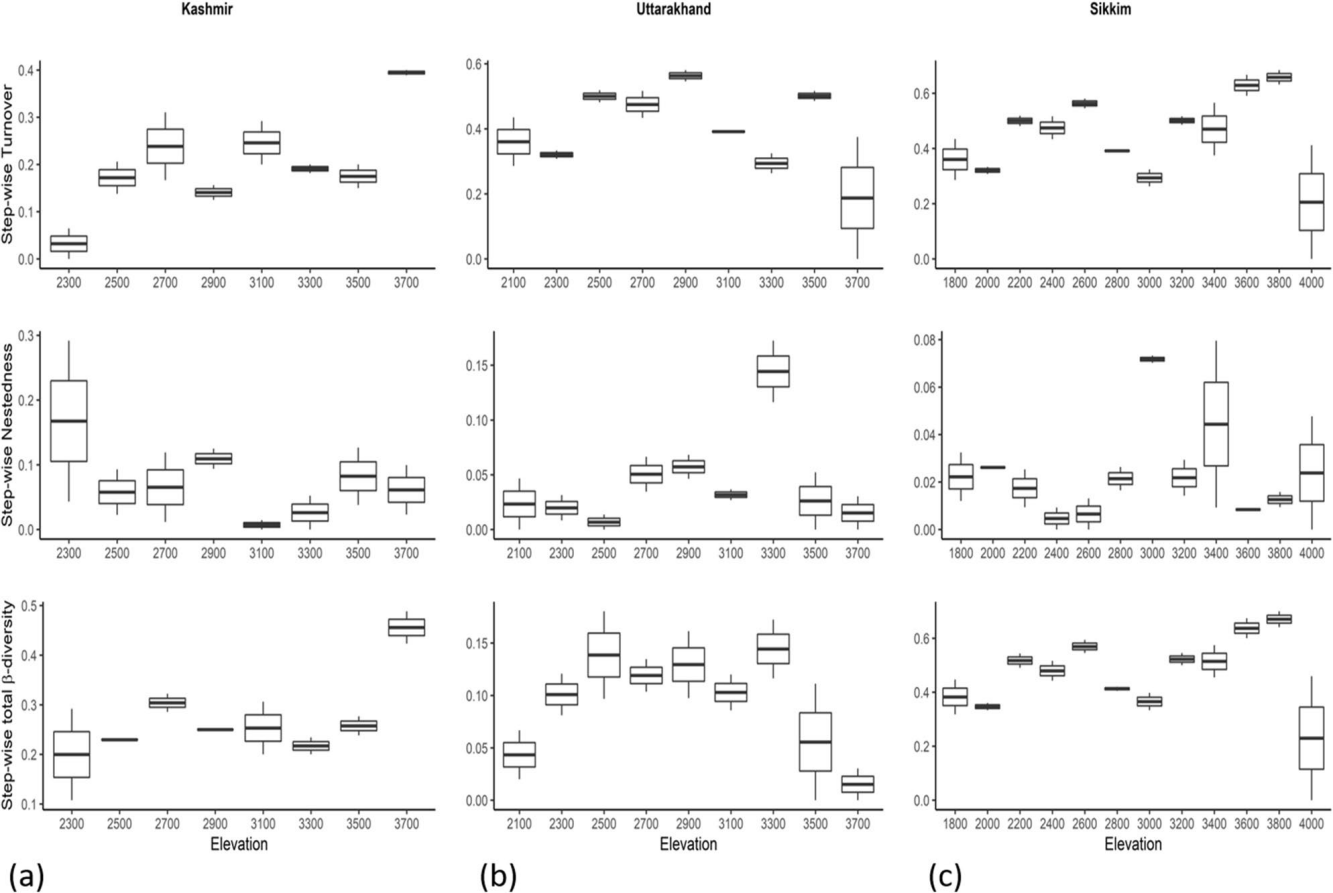
Stepwise turnover, nestedness and total β-diversity (pair-wise comparisons) of adjacent elevations in **(a)** Kashmir, **(b)** Uttarakhand and **(c)** Sikkim (the elevation band represents its comparison with adjacent elevation bands below and above it).

The comparison of turnover, nestedness and total β-diversity in an elevation band with adjacent elevation bands below and above it (Fig. 11) revealed that the turnover in the Kashmir Himalayan transect was low in the lowest elevation band, was higher in middle elevation bands and reached the peak in the highest elevation band. Turnover in Uttarakhand and Sikkim transects was higher in low and mid-elevations but declined in the highest elevation band. Nestedness, in the Kashmir Himalayan transect, was more or less low across elevation bands except for a slightly higher value in the lowest elevation band. A similar trend of low nestedness was noticed in the Uttarakhand transect except for the elevation band of 3300 (m a.m.s.l.) where it was exceptionally high. The pattern of nestedness in the Sikkim transect was consistently low in lower elevation bands but showed variation beyond 3000 m elevation. Stepwise total β-diversity in Kashmir and Sikkim transects more or less increased with elevation but in the Uttarakhand transect, it showed a mid-elevation peak and then declined in higher elevations (Fig. 11).

## Discussion

The present macroecological study of macrolichen richness and diversity along elevational gradients across the Himalayan longitudinal arc revealed that the species richness and identity not only varied with elevation but also across the arc. Non-metric multidimensional scaling (NMDS), an indirect gradient analysis approach that produces an ordination based on a dissimilarity matrix, also confirmed that the three transects differed in species composition (Fig. 7) and differences were statistically significant (Table 3). It is quite expected as the three sites are spatially separated with marked differences in climatic conditions^45^ and also differ in the habitat-types that they offer for lichens^46,47^, such as tree bark types, soil type etc^47–49^. The three transects not only differed at the species level, but also at generic and family levels. However, the genus *Cladonia* and family Parmeliaceae, known for a wide tolerance range^49^, dominated across the studied transects (Figs. 5, 6). Parmeliaceae is the largest family world over representing 1/10th of lichen species^50^ and its members grow on all types of substrates in a wide range of climatic conditions^51^. Though the species assemblages were taxonomically different, they were more or less functionally similar with the dominance of foliose and corticolous lichens (Table 2). We argue that this may be due to the large share of forest area in each of the transects which provides broadly similar habitats for the functionally similar lichen species to grow. Species richness patterns along the elevation gradient were not consistent across different growth forms and habitat categories. From north-western Kashmir to eastern Sikkim, spline.plots revealed the most common hump-shaped distribution pattern in overall macrolichen richness (Fig. 8a) which is consistent with other studies across Himalaya for similar taxa^49,52,53^ and for other taxa such as vascular plants^54^, seed plants^55^, ferns^56^, bryophytes^57^. In Uttarakhand transect, however, a low plateau type pattern was reported that has often been reported in birds, reptiles and plants^58^. Among the growth forms, foliose lichens and among substrate categories, corticolous lichens also revealed the common hump-shaped elevation pattern in Kashmir and Sikkim but a low plateau pattern in Uttarakhand transect (Fig. 8b,c). The other growth forms and substrate categories of lichens that showed hump-shaped pattern with elevation include fruticose, squamulose and terricolous in Uttarakhand and Sikkim transects, respectively. A hump-shaped pattern in terricolous lichens along elevation gradient has been earlier reported in Garhwal Himalaya as well^53^. The hump-shaped distribution of foliose lichens may be due to their ability to utilize the light even at shady places of thick forests and on low canopy twigs^59^, and for fruticose lichens, mid-elevational pattern could be because of their tolerance to severe environmental limits, acidic soils and effect of grazing which is quite common at mid altitudes in the Himalaya^49,60^. Nonetheless, for corticolous lichens, this may be attributed to an increase in forest cover from low to mid-elevations, thereby offering suitable habitats for them and less suitable habitats for saxicolous lichens which show more or less increasing richness above the treeline ecotone due to an increase in rocky substrates^54,61^. Hump shaped pattern in the case of terricolous lichens may be due to a decrease in overall soil cover at upper elevations^49^. While inverse hump-shaped patterns were observed for crustose and saxicolous forms in Kashmir, some growth forms and substrate categories did not show any significant relationship with elevation (Fig. 8). The inconsistency in species richness patterns among various growth forms and substrate categories of lichens along the Himalayan longitudinal arc could also be due to differences in the climatic and other features across the arc^62,63^.

Unlike many previous reports^11,64^, total β-diversity increased significantly (Table 5) with elevation which was largely due to the turnover component (Fig. 9) and such an observation is consistent with several other similar reports^15,19,43,65^. An increase in total β-diversity and turnover with elevation in the three transects could be due to the restriction of particular lichen species to different elevation zones because of the availability of suitable habitat/substrate for these groups in these zones. Such distinct elevation zones, namely closed-canopy forest, treeline ecotone and treeline alpine tundra have been reported in the Himalaya^66^ and not many species occupy the entire elevation gradient. The restriction of species to these different zones may be largely due to environmental filtering which is primarily determined by evolutionary and historical factors and also by dispersal barriers^67^. Thus, species sorting and abiotic factors may be the main determinants of the lichen species replacements with elevation. Moving from Kashmir to Sikkim across the Himalayan longitudinal arc, the total β-diversity and its turnover component increased. β-diversity generally increases from temperate to tropical regions^64^. It may also be due to increasing physical and abiotic limiting factors associated with the gradient length which was lowest in Kashmir and highest in Sikkim. The nestedness (Fig. 9) varied independent of the gradient length with Kashmir and Sikkim transects showing significant decline in the nestedness with elevation (Table 5) but increase with elevation was noticed in Uttarakhand transect. These observations indicate that lichen assemblages at species poor elevations are not subsets of species that were recorded in rich elevation zones in Kashmir and Sikkim. It indicates that differentiation of lichen assemblages occurs at different elevations because of which the distribution range of various lichen species does not overlap. Consequently, the lichens that occurred at lower elevations were absent from higher elevation bands and such results have been reported earlier also^64^. Our results are in contrast to the usual pattern of decrease in the β-diversity and species turnover with increasing elevation. Nestedness, on the other hand, did not show a consistent pattern with elevation. It increased with elevation in Uttarakhand transect but declined with elevation in Kashmir and Sikkim transects. An increase in turnover, as well as nestedness as observed in the the Uttarakhand transect, has also been reported for grasshoppers^43^. In the case of stepwise comparisons of β-diversity, total β-diversity showed a significant relationship with elevation (p ≤ 0.05) in the Kashmir Himalayan transect and it was largely due to species turnover which also showed a significant relationship with elevation (p ≤ 0.05) unlike nestedness. In case of Uttarakhand and Sikkim transects all the attributes, including β-diversity, species turnover and nestedness did not show any significant relationship with elevation. Similar findings have been reported for several taxa^43^.

## Conclusion

Based on the present study, it can be concluded that the taxonomic composition of microlichens varied across the Himalayan arc, but this variation was not reflected in the richness of functional groups, as foliose, fruticose and corticolous forms were dominant in all three transects. Most of the lichen groups showed hump-shaped elevation pattern in species richness though an inverse hump-shaped pattern was also observed. β-diversity (β_sor_) varied across the transects but turnover was always the dominant contributor to β-diversity instead of nestedness indicating the role of environmental sorting or spatial and historical constraints in the replacement of some species by others. While stepwise β-diversity and its components of turnover and nestedness did not vary significantly with elevation except in Kashmir, along elevation β-diversity and its components of turnover and nestedness varied significantly in relation to altitude at all the three transects implying that diversification of microlichen assemblages occurs with increasing elevational distance.

## Supplementary Information

Supplementary Information.

## Data Availability

The macrolichen species recorded at the three transects during the present study are listed in the Supplementary Information file of this article.
